# Playing the long game: Exploring the phenomenon of dementia-friendly golf

**DOI:** 10.1177/14713012211019498

**Published:** 2021-05-14

**Authors:** Robbie S Norval, Fiona Henderson, Geoff Whittam

**Affiliations:** 70447 Glasgow Caledonian University, Glasgow, UK

**Keywords:** activity, carers, dementia, golf, qualitative study, social enterprise

## Abstract

As individuals age, participation in previously accessible leisure activities can be compromised through diminished capabilities and negative societal expectations. This study investigates the unexplored accessibility of golf for older people with dementia using interviews and observations of Scottish participants in social enterprise–led golfing activities. The resulting thematic analysis concluded that golf is an accessible activity for people living with dementia, and continued participation generates social connectedness and enhances well-being. However, there remain social barriers to participation including societal stigma surrounding the perceived abilities of people living with dementia and the perception of golf as a middle-class and male-dominated sport.

## Introduction

There are currently over 90,000 people who are living with dementia in Scotland and this number is growing (Scottish Government, 2017). Previous research has highlighted the positive impacts of older people’s participation in social enterprise-led activities, benefiting self-worth, identity and creating novel social opportunities (Henderson et al., 2020). Participation in leisure and physical activities is additionally supported by policy makers; the most recent Scottish Dementia Strategy advocates the use of therapeutic activities for people living with dementia (Scottish Government, 2017).

Leisure activities can provide wide-ranging benefits for people living with dementia with research highlighting promising findings on quality of life, reduced agitation, social inclusion and improved cognition (Cowl & Gaugler, 2014; Legere, 2018). A key component in facilitating effective activities for individuals living with dementia is ensuring that the activities are meaningful and create a connection with the participants (Han et al., 2016). Meaningful activities can provide an outlet for people living with dementia to reaffirm their identities allowing them to focus on their skills and abilities, enhancing their sense of personhood (Genoe & Dupuis, 2011). Tailoring interventions to the skill set and personalities of people living with dementia can also create more meaningful experiences by uncovering deeper aspects of their identities, invoking memories of previous roles and habits (Evans et al., 2019; Gitlin et al., 2008). In addition to adapting activities to the needs and wishes of the participant, environmental and pragmatic factors should also be considered to successfully facilitate meaningful interventions (Dugmore et al., 2015).

Despite findings highlighting the positive outcomes of dementia-friendly interventions, people living with dementia often need to overcome societal barriers to be able to participate in activities. The majority of respondents from a public survey in the United Kingdom felt that older adults would not be able to continue to participant in hobbies if they had a dementia diagnosis (Kane & Cook, 2013). Such generalisations debase the wide-ranging capabilities of people living with dementia, failing to recognise their individual strengths and abilities (Mitchell et al., 2013). Public perception also affects the social confidence of individuals living with dementia who often disengage from activities for fear of making mistakes or embarrassing themselves (Caddell & Clare, 2011; Roland & Chappell, 2017). To help break down these barriers, activities should be made as inclusive and accessible as possible to support people living with a dementia diagnosis (Fortune & Mckeown, 2016).

Golf is an activity favoured by older adults, which is physically more accessible than other forms of sports and importantly can still be played later in life, providing physical and cognitive health benefits as well as increasing the social circles of the participants (Stenner et al., 2016). In recent years, the phenomenon of dementia-friendly golf has become more prominent as golf clubs seek to make the game more accessible for their diminishing and ageing memberships (McLaughlin, 2019). However, despite the contemporary emergence of this phenomenon, research has neither explored the practical feasibility of these interventions nor the impact of such programmes for people living with dementia. This study aims to address these gaps by exploring the viability and impacts of golf as a dementia-friendly activity.

### Background information

‘Golden Golfers’ is a social enterprise which offers dementia-friendly golf sessions from different locations across Great Britain. The guiding aim of the organisation is to make golf more accessible regardless of age or skill with particular focus towards people living with dementia or Parkinson’s. This study took place at one of Golden Golfer’s partner golf clubs which was located in Glasgow. The group sessions were held on a weekly basis and lasted from 10am to 12.30 pm During the sessions, staff members (golfing buddies) were paired with participants to assist them in playing golf, providing companionship and support during the activity. After playing, participants and staff members came back together to have hot drinks and a bite to eat in the clubhouse.

### Methodology

This exploratory study adopted a qualitative approach to develop an understanding of the lived experiences of people living with dementia playing golf. A total of 12 people were interviewed for this study, 3 participants living with dementia, 3 carers, 1 older adult participant and 5 staff members from the social enterprise. Ethical permission was granted from the University’s Ethics Committee in August 2019, and informed consent was obtained from all the participants involved. Data were collected between November 2019 and September 2020, and due to the COVID-19 pandemic, the data collection was suspended from mid-March until June 2020.

Purposeful and volunteer sampling were utilised in the selection of participants for this study. We purposefully selected 5 staff members to be interviewed: the founder, project manager, the facilitator and 2 golfing buddies to provide a range of perspectives from within the organisation. The facilitator of Golden Golfers then approached players individually inviting them to be involved in the study. Seven volunteer participants who expressed an interest were subsequently interviewed. Participants living with dementia either self-identified as living with dementia or their carers informed us about their diagnosis.

The semi-structured interviews were guided by a set of core questions which took two forms. Firstly, questions were designed for staff members regarding the planning, facilitation and social impact of the sessions. Questions ranged from ‘*What practical considerations are made to meet the needs of participants living with dementia*?’ to ‘*In your opinion, what is the impact of the sessions for participants?*’. Secondly, questions were designed for participants and carers. These questions focused on their motivation and experiences in attending the sessions. The questions varied from ‘*What was your initial motivation in attending Golden Golfers*?’ to ‘*How do you feel after attending the sessions?*’. Participants living with dementia were interviewed alongside their partners to make the interviews less intimidating. Interviews lasted on average for 30 min, ranging from 25 to 45 min. The interviews were audio recorded and transcribed verbatim to verify that the data were accurate and accountable.

In addition to the interviews, this study included 12 h of observations. We utilised a participant as observer stance observing the activities and interactions between participants and staff members on the driving range, on the course and in the clubhouse (Gold, 1957). During the observations, we played some golf and assisted participants with carrying golf clubs. Brief field notes were written on a notepad on site before being expanded upon after the completion of the interventions.

During the interviews and observations, participants living with dementia often expressed themselves with one-word answers, broken or sometimes incomplete sentences. We have therefore incorporated the meaning of their verbal exchanges and non-verbal expressions through written narrative as opposed to direct quotes to make sure that the contributions of participants living with dementia were included and represented throughout this study. All the direct quotes used in the findings of this study derived from the semi-structured interviews, while the contextual data stemmed from both semi-structured interviews and observations. The data were anonymised by removing the real names of the organisation and the participants replacing them with pseudonyms. After the collection of data, an inductive thematic analysis applying Braun and Clarke’s approach was utilised before the findings were written up (Braun & Clarke, 2006).

Due to the involvement of people living with dementia, considerations were made regarding the accessibility of this study to include and safeguard participants throughout. We utilised the process consent procedure, designed specifically to continually obtain and monitor consent for people living with dementia (Dewing, 2007). We also provided participants with information designed in a dementia-friendly format and sought consent from both the carer and person living with dementia before the data were gathered (Dementia Engagement and Empowerment Project, 2013).

## Findings and discussion

The analysis found three emergent themes from the data collection, namely (1) *TaylorMade* (bespoke golf sessions), (2) *The 19th hole* (social opportunities) and (3) *From bogey to birdie* (overcoming stigmas).

### TaylorMade^1^ (bespoke golf sessions)

An important component of Golden Golfers was making their services accessible for older adults and people living with dementia. All of the participants were golfers who felt they could no longer continue to play at their local golf courses because a full round of golf was too physically demanding. Accessibility of golf was therefore a prime consideration for Golden Golfers. The ethos of the social enterprise was that anyone, regardless of their golfing skills, physical or cognitive capabilities, would be welcomed. These supportive attributes were instilled in the services offered by the organisation as explained by the founder Thomas:

*“We give people every chance to be Tiger Woods, you know, if that's what they want to be. But if they're not going to be Tiger Woods, and they'll never go beyond the putting green and the coffee bar, then we can facilitate that as well in our sessions. So their journey goes at their speed with our support. It can go as fast or slow as they want it to”* Thomas (staff member).

The organisation took practical measures to provide a bespoke service for their participants. This was achieved by breaking down the game into accessible parts including reserving set tee times, playing a few holes, hitting some balls at the driving range, mini competitions on the putting green and indoor golf quizzes if bad weather prevented them from going out on the course. An accommodating approach was implemented throughout the duration of the sessions as participants were paired up with a buddy who provided them with advice and physical support on the golf course as Felicity explains:

*“Perhaps Hannah would play all her own shots because she was very capable, and her golf was still at a good standard whereas Pamela couldn't really hit a long ball very well. I would join up with Pamela, and I would hit the long shots. Then when we got nearer the green, she would do the chipping and the putting, so really just tailoring it to the participants' needs”* Felicity (staff member).

Alongside the physical and pastoral support, staff members within the organisation implemented a personalised approach in delivering their sessions considering health, well-being and environmental factors before starting play:

“…*you know with a dementia journey, things change, so I guess what we're trying to do is assess, trying to think right today might not be a good day for Toby to be out golfing, for example, his medications just changed. And we've heard from his wife that he's been a bit unsteady on his feet. So let's review, re-evaluate what we're going to do with him today*” Julianna (staff member).

The personal touches and tailoring towards the needs and wishes of their participants increased the confidence and well-being of the participants (Evans et al., 2019). Kitwood (1997) advocates the importance of personhood and recommends building up meaningful relationships with people living with dementia to gain a deeper understanding of their traits and wishes. The concept of personhood was prominent throughout the observations as Golden Golfer’s bespoke approach enabled participants regardless of physical or cognitive impairments to continue to play golf later in life.

### The 19th hole (social opportunities)

The term “the 19th hole” was internally used within the organisation to describe the social element of the activity and was a key finding from interviews with participants, carers and staff members.

Five out of the seven participants and carers spoke about reduced social options for older adults and talked openly about friends and family who had passed away and the impact that had on their social lives. Diminishing social opportunities in older age is well established within academic literature; as physical and cognitive health deteriorate, social opportunities begin to shrink increasing feelings of loneliness (Urbanska et al., 2015). To counteract this, Golden Golfers sought to increase social opportunities by reserving a social space in the clubhouse where everyone could come together after playing golf:

*“When their husbands, wives, or carers came back, and we were all sitting having coffee, they would sit down with us and the stories would be regaled again of what a great putt Bettie had had. It was just really nice for them to see their loved one having a really nice relaxing time so the whole mood just lifted”* Felicity (staff member).

As expressed by Felicity, the social catch up facilitated a platform where participants, carers and staff members could recount their golfing highlights. Academic literature advocates the importance for people living with dementia to experience positive emotions and to undertake activities which bring them joy (Genoe & Dupuis, 2011). Additionally, research conveys the need for people living with dementia to possess different roles within society and not be defined by a dementia diagnosis; within the context of this study, the participants possessed different roles as golfers, learners and friends (Genoe & Dupuis, 2011). Golden Golfers therefore facilitated new opportunities for people later in life, enabling relationships to be built, expanding the social circles of their participants. A typical viewpoint was:

*“I was wandering about like a lost soul therefore I was actually delighted to be able to come and meet people, now I speak to Julianne regularly and Paul, all because of Golden Golfers”* Tommy (participant).

As well as social benefits for participants, carers spoke about how Golden Golfers built up their own personal network:

*“There's always lots of chat and lots of laughs. Actually, meeting Cameron's wife Jolene has been very good for me because she's in a carers groups and she's given me a lot of useful information that I can use as well”* Kirsty (carer).

As articulated by Kirsty, the social elements of Golden Golfers went further than simply connecting participants, but instead created a wider community where carers could increase their knowledge and awareness of the local dementia landscape. Every participant, carer and staff member spoke about the social benefits they experienced through participating in Golden Golfers; therefore, this theme reflects the overarching essence of the organisation.

### From bogey to birdie (overturning stigmas)

The prevalence of stigma was a common theme within this study, taking two different forms: firstly, stigma attached to a dementia diagnosis and secondly, stigma surrounding the perception of golf. Two-thirds of interviewees believed that societal barriers existed about golf being a suitable activity for people living with dementia:

*“Golden Golfers have got a difficult job, a very difficult job, I see what happens whoever has the dementia, the wives are saying there is no point taking them up (to the golf)”* Tommy (participant).

In addition to this perception that golf would not function as a suitable activity for people with a dementia diagnosis, participants described prior personal experiences which affected their ability to golf. One golfer recounted that she started to forget her score and was accused of cheating by other players. Fellow members no longer wanted to play with her, alienating her from previous contacts and friends. This feeling of being ostracised is unfortunately often reported by people living with dementia as friends and acquaintances can be unsure about how to act, which can lead to them distancing themselves (Urbanska et al., 2015):

*“I know it's not their fault (members of the public), because I know some people are a bit scared of dementia. They think "How do I treat these people?" They're not "these people". They are flesh and blood like anybody else. They're still themselves”* Kevin (staff member).

As voiced by Kevin, respect and dignity were encompassed in the social enterprise’s core values. This inclusive environment created a safe space for people living with dementia, enabling them to continue their pastime free from societal preconceptions or judgement. The sessions also allowed participants to continue to play active roles in their communities increasing their profiles as capable citizens. These findings spark further discussions about how leisure can be used to overcome stigma. If participants living with dementia can resist stigma and are able to participate in activities, they can challenge set societal views (Genoe, 2010). By overcoming prejudices through leisure activities, people living with dementia can also possess greater agency by making decisions and expressing a range of emotions (Genoe, 2010). Using leisure as a platform for resistance against dementia-based stigmas also has practical considerations for activity providers. It may encourage them to reconsider their offerings ensuring that they are providing engaging, inclusive and novel activities and not reinforcing negative ageing stereotypes (Dare et al., 2018; Fortune & Mckeown, 2016; Genoe, 2010). In addition to dementia-based stigma, the staff members were also conscious of how golf was perceived externally. Andrew expressed concerns about the socio-economic demographics surrounding golf:

*“There is sometimes a view of golf and I think we're a victim of that sometimes as well. Golf is seen as elitist and that still can be the perception”* Andrew (staff member).

Participants and staff members spoke about gender-based biases which existed within the golfing community as touched upon by the facilitator Julianne:

*“Challenges, I suppose include awareness raising as well, and especially even the perception of golf. When you think about golf, it has the reputation for being quite sexist”* Julianne (staff member).

Academic and grey literature mirror Andrew’s and Julianne’s statements underlining that golf is often perceived as an elitist and male-dominated sport (Mcginnis et al., 2005; Peppard, 2016; Stenner et al., 2016). Staff members were conscious of such associations and made efforts to engage and promote the social enterprise to show that their services were universal and inclusive, challenging fixed mindsets that golf was prestigious and exclusive. Therefore, although internally Golden Golfers offered a very inclusive and accessible service as testified by all participants and carers, external stigmas regarding elitism and sexism adversely affected their ability to attract new golfers. This was further compounded by disempowering societal opinions and a lack of social awareness regarding dementia which impacted on Golden Golfer’s ability to engage with new participants living with dementia.

### Limitations

As this is an exploratory study, it is a relatively small sample size and has limitations in terms of its homogeneous nature. Future research may look to explore the impact of golf for people living with different types of dementia incorporating participants who are at different stages of their dementia journey.

## Conclusion

These findings suggest that stigma exists regarding what activities are deemed appropriate for people living with dementia and demonstrate the societal barriers in place for individuals continuing to participate in hobbies with a dementia diagnosis. The research, however, also portrays a positive message highlighting the viability of golf as a dementia-inclusive activity. If person-centred and accessible support is provided, then golf can serve as an activity that can be continued to be played by people living with dementia. Furthermore, golf can also be used as a platform to organically establish new social opportunities for both people living with dementia and their carers increasing their respective social circles. These findings also have practical considerations for golf clubs suggesting that working in partnership with dementia-focused organisations, increasing internal awareness and providing space and support for people living with dementia can enhance their golfer’s experiences and create a more accessible and inclusive service for their ageing memberships.

## Note

1. *TaylorMade as opposed to Tailor Made, it is intended to be a play on words as TaylorMade is a famous golf brand.
